# Effect of Chemical Admixtures on Mechanical and Degradation Properties of Metallurgical Sludge Waste Concrete

**DOI:** 10.3390/ma15238287

**Published:** 2022-11-22

**Authors:** Petr Lehner, Marie Horňáková, Jan Pizoń, Jacek Gołaszewski

**Affiliations:** 1 Department of Structural Mechanics, Faculty of Civil Engineering, VSB-Technical University of Ostrava, Ludvíka Podéště 1875/17, 708 33 Ostrava, Czech Republic; 2 Department of Building Processes and Building Physics, Faculty of Civil Engineering, Silesian University of Technology, Akademicka Street 5, 44-100 Gliwice, Poland

**Keywords:** aggregate replacement, concrete, metallurgical sludge waste, electrical resistivity, compressive strength

## Abstract

This study extends the development of concretes with metallurgical sludge waste (MSW) by determining the effect of superplasticizers and air entrainment admixture (AEA). The MSW is a very fine powdery material, and in this case, it was used as a partial replacement of fine aggregate in the mixture. The reference ordinary concrete mixtures without MSW were created for evaluation. The results of concrete density, compressive strength, electrical resistivity, and rapid chloride permeability were obtained and some of them were measured continuously to determine the influence of the chemical admixtures on these characteristics over time. It was found that in general, the MSW substitution slightly lowers the mechanical and durability parameters, but MSW in combination with the chemical admixtures improves the compressive strength in comparison to the reference concrete with the addition of AEA.

## 1. Introduction

Sustainable development of the construction industry, and specifically the concrete industry, requires innovative and unconventional approaches [1]. One of the many problems in the concrete industry is the high environmental impact of natural aggregates obtained by the process of traditional mining, which is also very expensive [2]. It is, therefore, necessary to look for alternatives of aggregates that are both suitable for concrete production and environmentally friendly as well. It is currently common in practice to use waste concrete from construction demolitions for this kind of purpose [3]. Many research groups dedicated their projects to examining the possibilities of using other waste materials, such as red ceramic debris [4], sanitary ware [5], tires [6], plastic bottles [7], and many others. However, suitable waste material for concrete production does not have to come only from the construction industry. Metallurgical waste from industrial areas, such as sludge (MSW), slag, and dust from blast furnaces, steel, and sintering processes, is a typical example of this material. The metallurgical waste material is stored as a by-product in large landfills and there is also a danger of hazardous compounds leaking into the ground and groundwater [8]; it is, therefore, in the best interest to study its applicability in different industries. Research on the usability of MSW is already underway around the world [9,10]. In 2019, approximately 1300 Mt of metallurgical waste was produced worldwide. Its use is restricted due to its qualification as hazardous waste, but this is not an end state that we cannot change [11]. In this study, MSW was used as a partial replacement for fine aggregate in concrete. Although MSW is a generally known material, its use as a component of concrete is not widely researched. Experiments on mechanical and rheological properties were carried out in [11,12]. However, research on a broader scale of properties, especially long-term and durability ones, still needs to be executed. The mechanical and degradation properties of concrete mixes are influenced not only by the composition but also by chemical additives. Admixtures allow beneficial modification of the properties of the concrete mixture and/or hardened concrete. As a result, it is possible to design concrete structures with high strength, environmental resistance, and architectural qualities and, what is particularly important, to execute these structures even in case of complicated requirements and/or difficult technical conditions. In addition, by using admixtures, it is possible to achieve very significant improvements in the working conditions for concreting. In our case, the use of a superplasticizer allows us also to compensate for the negative effects of metallurgical waste on the workability of the concrete mix. In this case, four concrete mixes were analyzed to determine the effect of MSW, superplasticizer, and air-entraining admixture (AEA) on the material characteristics. Two of them are considered reference mixes without MSW and in two of them, the MSW was used as a 30% replacement of fine aggregate. Superplasticizer needed to be applied in both MSW concrete mixes because it helps to achieve the required consistency of the mix. The AEA is usually used in concrete mixtures to increase the aeration of the mix for better workability and higher frost resistance [13]. On the other hand, in some cases, AEA had a negative effect on compressive strength and other degradation processes in concrete [14]. The results are used to evaluate the effect of these chemical admixtures on the mechanical and degradation properties of concretes with MSW. The effect of AEA in combination with MSW on mechanical and durability parameters has not yet been demonstrated and this is the main contribution of the paper presented here.

## 2. Materials and Methods

The aim of the whole research is to evaluate the usability and effect of MSW on the mechanical and durability properties of the final concrete mixes to create more sustainable concrete and used the waste product, which would otherwise just clutter the landfill. The MSW was considered a replacement for a fine aggregate since by its nature it resembles it. In this study, the concretes were also enriched by chemical admixtures (superplasticizer and air-entraining admixture) to determine their influence on the mentioned properties, which were measured at specific time points to properly capture their effect.

### 2.1. Metallurgical Sludge Waste

The metallurgical sludge used in the research was obtained from a Polish steel producer. It consists of agglomeration sludge, blast furnace sludge, and converter sludge in different proportions. The processed MSW is a very fine powdery material consisting of grains of 0–0.25 mm in size. The chemical composition of MSW (see Table 1) depends on the exact point of the landfill and it was tested and described in detail in [12]. The reactivity of MSW was tested in [8] by microcalorimetry method and no evidence of exhaled heat during the reaction with water was observed. The zinc content of MSW needs to be taken into account to use it as an inert filler of concrete because it leads to retardation of cement hydration reaction [15].

### 2.2. Concrete Compositions

Compositions of concrete mixes are given in Table 2. Portland cement (CEM I 42.5 R), water, and natural aggregate (coarse and fine) were used. Natural gravel was used as a coarse aggregate and natural quartz sand was used as a fine one. Aggregate mix was composed of three separate fractions 0–2 mm (25%), 2–8 mm (35%), and 8–16 mm (40%).

A “reference” mixture containing neither MSW nor other chemical additives was prepared as a base one. The “MSW” mix was prepared with the 30% fine aggregate substitution by MSW. This mix had to be enriched with a superplasticizer because MSW mixes generally have a higher water consumption. Another reference mix (“Ref + AEA”) was prepared and enriched with AEA. As the last mixture (“MSW + AEA”), a combination of the fine aggregate replacement by MSW and addition of superplasticizer and AEA was prepared.

### 2.3. Samples Preparations

All concrete mixes and specimens were prepared and cured in the same way. The mixes were poured into molds immediately after mixing and demolded after 24 h. The storage of all samples was carried out under the same standard laboratory conditions in water storage tanks with regular water. Concreting and testing of mechanical properties were carried out at the Silesian Technical University in Gliwice; testing of degradation parameters was completed at the VSB-Technical University of Ostrava.

**Figure 1:**
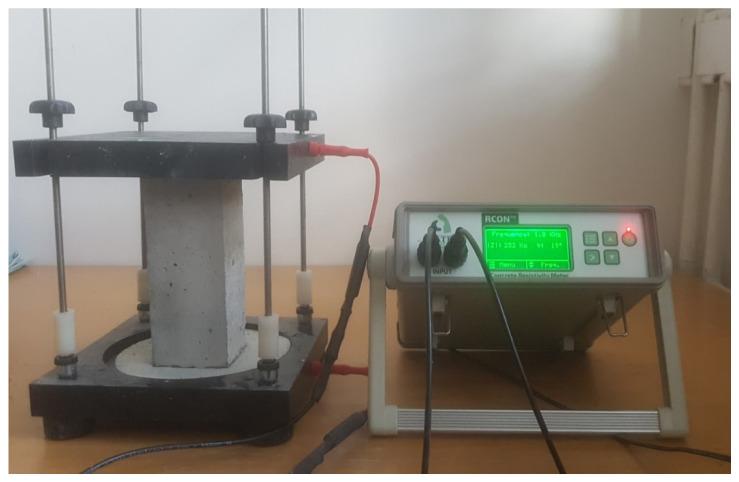
Prismatic sample placed in the RCON instrument.

Three specimens of every shape were cast from each mix: in the form of a cylinder (Ø100 mm × 200 mm) and a prism (100 mm × 100 mm × 200 mm—see Figure 1) for electrical resistivity testing (surface and bulk resistivity), a cylinder (Ø100 mm × 200 mm) for rapid chloride permeability testing, and a cube (150 mm × 150 mm × 150 mm) for compressive strength testing. For compressive strength testing, nine specimens were prepared for each mix so that testing could be performed at three times from the time of concreting. The other experiments are non-destructive, and therefore, the same specimens could be used to measure the values in time. The specimens used for non-destructive experiments were stored in water tanks also between the measurements. For every type of mixture, 18 specimens in total were created for the measurements.

### 2.4. Experiments

The experimental program was designed to evaluate the effects of chemical additives on mechanical and degradation parameters. The objective was to determine whether deterioration or improvement of properties happens and to what extent is caused by substitution of fine aggregate by MSW or by the addition of superplasticizer and AEA. The changes in properties over time caused by different concrete maturation were analyzed as well.

#### 2.4.1. Density

The density of hardened concrete was tested according to EN 12390-7 [16] at 28 days after concreting. The test specimens were weighed, and their exact dimensions were measured. Based on the measurements, the density was calculated, and the mean and standard deviation were determined using generally known statistical methods.

#### 2.4.2. Compressive Strength

Compressive strength was measured according to EN 12390-3:2011 [17]. Three specimens with dimensions of 150 mm × 150 mm × 150 mm were used and prepared in three batches. The reason was to obtain values of compressive strength at 28, 56, and 91 days after concreting using this destructive method. Therefore, the trend of the medium-term maturation of the concrete can be evaluated and, in addition, the results can be correlated with other non-destructive tests.

#### 2.4.3. Electrical Resistivity and Diffusion Coefficient

The surface resistivity was measured by the Wenner probe according to the AASHTO T358 standard [18] on every length side of the specimen. An RCON instrument [19] was used to determine the bulk resistivity according to ASTM C1760-12 [20] (see Figure 1).

Measurements were taken on three cylindrical and three prismatic specimens—at a time of 56 days after concreting. It was therefore possible to evaluate the influence of the mix composition on the results of the methods, which should produce the same results.

Due to the nature of the non-destructive testing of the method [21], it was possible to perform measurements at several times: 28, 56, 91, 112, 168, and 224 days after concreting. The results could thus be used for direct comparison with other tests performed at the same time. Another advantage of this non-destructive method is the possibility to calculate the diffusion coefficient as well as the maturation coefficient. The diffusion coefficient of chloride in concrete was calculated by the Nerst–Einstein Equation (1) [22], which expresses the relationship between electrical resistivity and diffusivity:

(1) $$D=\frac{RT}{Z^{2}F^{2}}\cdot \frac{t_{i}}{\gamma_{i}C_{i}\rho_{BR}}$$

where *D* is the diffusion coefficient (m^2^·s^−1^), *R* is the universal gas constant (J/K·mol), *T* is the absolute temperature (K), *Z* is the ionic valence (-), *F* is the Faraday constant (C/mol), *t*_i_ is the transfer number of chloride ion (-), γ_i_ is the activity coefficient of chloride ion (-), *C*_i_ is the concentration of ions i in the pore water, and ρ_BR_ is the bulk electrical resistivity (Ωm).

The *m*-factor, which describes the reduction of diffusion coefficient over time, was calculated by approximation of Equation (2) by the least squares method [23].

(2) $$D_{c,t}=D_{c,ref}{\left(\frac{t_{ref}}{t}\right)}^{m}$$

where *D_c_*_,*t*_ is the apparent diffusion coefficient for a selected age (m^2^·s^−1^), m is the aging factor (-), *D_c_*_,*ref*_ is the reference diffusion coefficient (m^2^·s^−1^) in the reference time *t_ref_* (years) and *t* is the specific time (years).

#### 2.4.4. Rapid Chloride Permeability Test

The rapid chloride permeability test (RCPT) according to ASTM C1202 [24] was used as an additional destructive test. By this method, it is possible to quickly determine the resistance of the concrete sample to chloride ion penetration. The test is performed by placing a 100 mm diameter and 60 mm long concrete cylinder in a device in which a 3.0% salt solution and a 0.3 N sodium hydroxide solution are prepared.

The sample was prepared by cutting one of the cylinders. Throughout the test, a voltage of 60 V DC is maintained at the ends of the sample and the charge that passes through the sample is recorded. Based on the charge, the permeability of the concrete can be qualitatively evaluated. According to the standard, the following chloride ion penetration limits are defined: >4000 C = high, 2000–4000 C = moderate, 1000–2000 = low, 100–1000 = very low, <100 = negligible.

## 3. Results

Some of the experiments were performed in various time points so that the maturation of the concrete could be evaluated and some of the tests were carried out in certain time sections only. For the destructive experiment of measuring the compressive strength, three time points of testing were prepared (28, 56, and 91 days after concreting). The other destructive RCPT test was carried out 56 days after concreting in order to assess the relationship with other tests. The measurements for the non-destructive tests (surface and bulk resistivity) were made continuously. The density of the specimens was determined only once, 28 days after concreting, with the assumption that it does not change over time. The chapter presents partial results first and then places them in the context of the time of the test.

### 3.1. Compressive Strength

**Figure 2:**
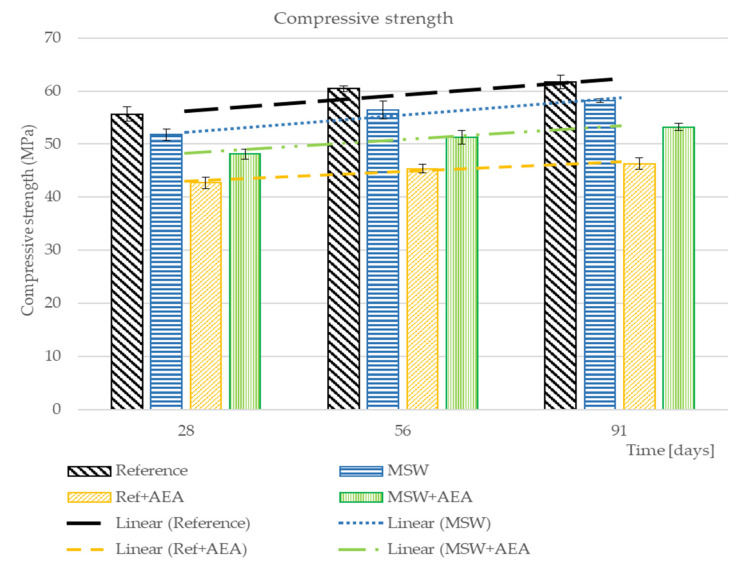
The values of compressive strength over the time.

The compressive strength results are shown in Figure 2. The differences between the strength parameters of every concrete at three different times are very similar.

The increase in strength is gradual, irrespective of the composition of the mix. Thus, MSW does not significantly improve or deteriorate the progress of the strength properties.

However, the lowest value of strength was achieved by the reference concrete with AEA. In contrast, the combination of MSW and AEA can slightly increase the strength. This phenomenon is caused by the different reaction of AEA with the replacement of fine aggregate. Further evaluation of compressive strengths is presented in the following chapters together with other results. It is a material with no significant chemical activity when in contact with water. Undoubtedly, the presence of MSW makes it necessary to use more superplasticizers and/or AEA to achieve a certain effect. This is mainly physical, due to the high water demands of MS, which is much higher than sand (and also cement, but in the research, MS was used as a sand replacement). MSW is very fine and contains wastes absorbing water. Some chemical interactions between MSW and admixtures cannot be ruled out, but this requires further research.

### 3.2. Diffusion Coefficient

**Figure 3:**
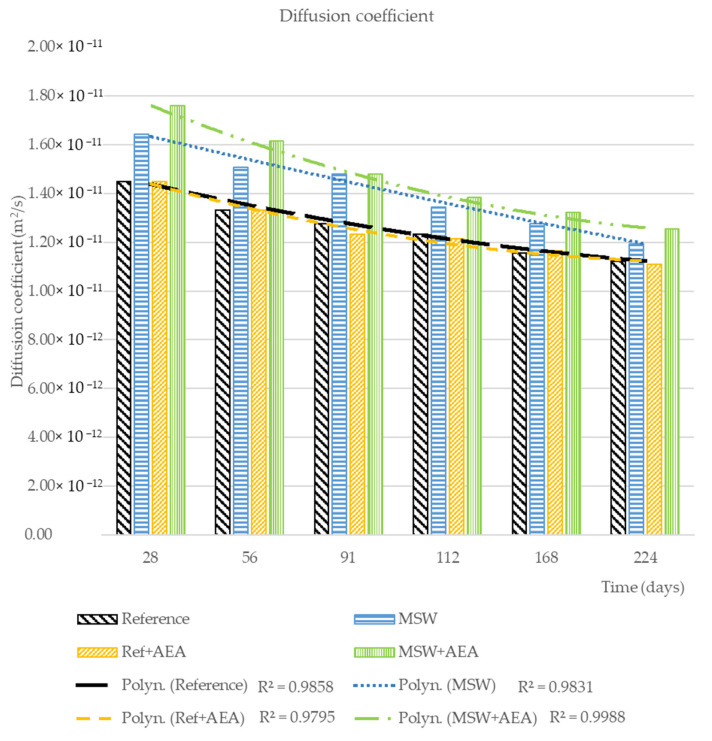
Diffusion coefficient over the time.

The diffusion coefficient at different times is a result calculated from the electrical resistivity, and it is an ideal parameter for the purpose of evaluating concrete degradation. The lower the diffusion coefficient is the higher the durability of concrete in an aggressive environment. Figure 3 shows the results for all the selected time points since the concreting.

The fundamental observation is that all concrete mixes show a decrease in the diffusion coefficient, i.e., an improvement of this characteristic, which indicates the resistance to penetration by aggressive substances. It is also possible to determine how the individual chemical admixtures or the MSW itself influence the resistance. A minimal difference can be seen in the case of the reference concretes. However, a much larger difference can already be observed in the case of MSW concretes; the resistance of the MSW concrete without AEA is higher than with AEA. However, the fundamental observation is that the initial diffusion coefficient values of MSW concretes are higher than the diffusion coefficients of the reference concretes but decrease more rapidly over time than the reference ones. Further evaluation of the electrical resistance is presented in the following chapters together with other results.

### 3.3. Comparison of Results in 28 Days

The main intention of the study was to evaluate the effect of superplasticizer and AEA on the mechanical and degradation behavior of concretes admixed with partial replacement of fine aggregate by MSW. Therefore, the results were grouped according to the time of testing after concreting. Three groups were created that contain different types of results, but always include all types of concrete mixes. The first group summarizes the results 28 days after concrete preparation, which includes density, compressive strength, and bulk electrical resistivity on cylinders (see Table 3).

**Figure 4:**
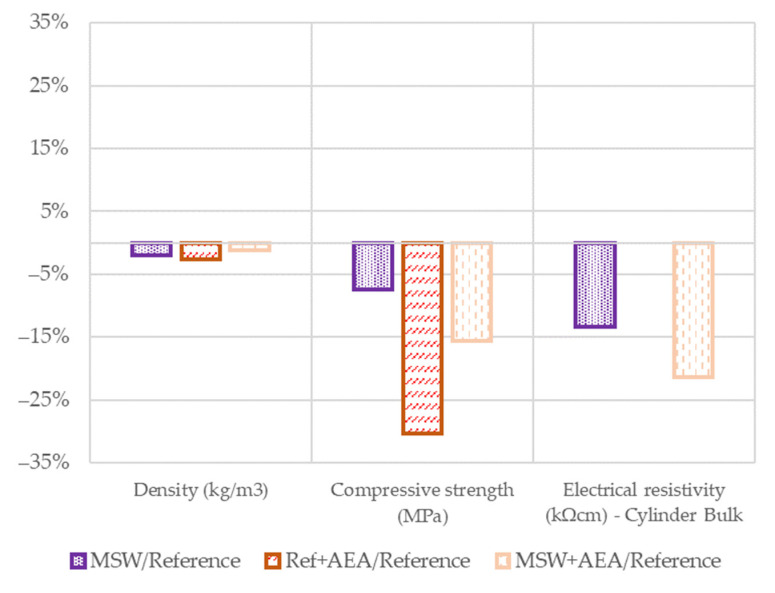
Relative results in 28 days.

To analyze the relative influence of the fine aggregate substitution and the chemical additives alone, the relative differences to the reference mixture were calculated. Thus, the absolute values of the results for the MSW, Ref + AEA, and MSW + AEA mixes were divided by the absolute values of the reference mix. Figure 4 shows the percentage values.

For density, the differences are minimal not exceeding −3%, which also shows the similar pore composition in the concretes. On the other hand, the compressive strength is 30% lower when AEA is added to the reference concrete. The concrete with MSW alone without the aeration admixture has a 7% lower compressive strength than the reference one and the addition of AEA further reduced the overall strength by 16%; however, this loss is not as significant as the reference mix. Electrical resistivity, which is one of the indicators of resistance to aggressive substances, shows zero difference for the reference mixtures. On the other hand, MSW mixtures show lower electrical resistivity by 13% and 21%, respectively, and thus worse resistance.

### 3.4. Comparison of Results in 56 Days

The second group of results is related to the time of 56 days after concreting. It contains a larger number of experiments: compressive strength, electrical surface resistivity on a cylinder and prismatic specimen, bulk resistivity on a prismatic specimen, and RCPT (see Table 4). All RCPT values are in the “moderate” chloride ion penetrability category.

**Figure 5:**
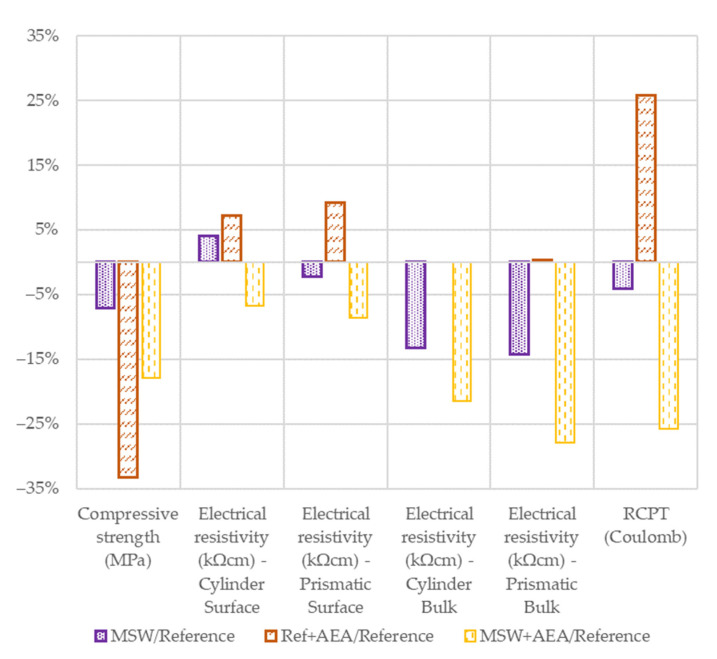
Relative results in 56 days.

Figure 5 evaluates the relative differences between concretes and the reference concrete mix.

For the compressive strength, the differences are almost identical after 28 days from concreting. However, the relative results of electrical resistivity, which was measured by two different instruments on two different shapes of the specimen, are not the showing the same trend. Even after taking into account the possible measurement error, the same trend is observed only in the case of the MSW + AEA mix, i.e., lower electrical resistivity from 7 to 28% than the reference values.

The Ref + AEA concrete also shows fluctuations on the positive axis, which may be caused by the non-uniform pore distribution over the surface of the samples. In general, the bulk measurement is more suitable for laboratory experiments and should be inclined to. Small differences in surface resistivity are often caused by the smooth surface of the sample, even if the internal structure is porous. Interestingly, the RCPT results have the same trend as the surface electrical resistivity for the Ref + AEA mixture, but also have a similar trend to the bulk electrical resistivity for the MSW + AEA mixture. From these data, one could infer the influence of the additives, which is consistent with the findings in the literature, for example [25].

### 3.5. Comparison of Results in 91 Days

The last group of results is evaluated at 91 days after concreting, which is the time when the concrete properties are expected to stabilize. Two experiments were carried out, namely the measurement of compressive strength and bulk electrical resistivity on the cylinder (see Table 5). 

**Figure 6:**
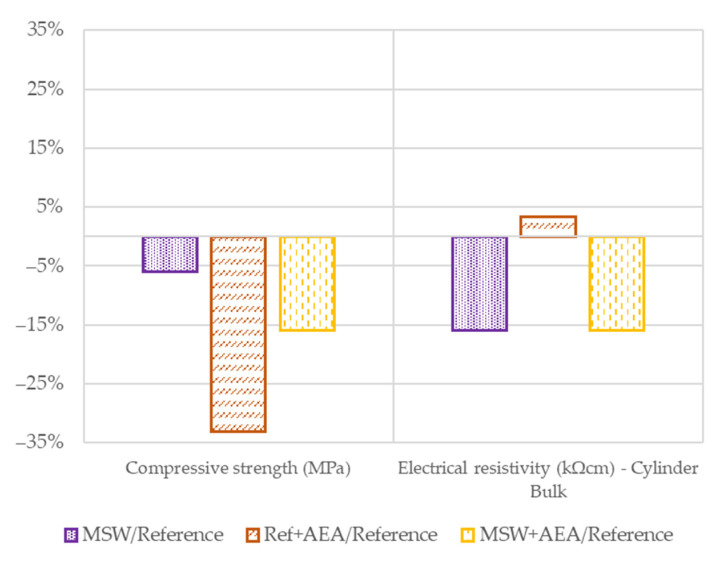
Relative results in 91 days.

Figure 6 presents the results of the relative differences compared to the reference concrete.

For compressive strength, the differences are again almost the same as at the previous times. Similarly, the electrical resistivity shows almost identical results as at 28 days but differs slightly from the results at 56 days—this may be caused by the allowable error in the measurements. In general, the same conclusions can be drawn as in Section 3.1 and Section 3.2.

## 4. Conclusions

The study presents the mechanical and degradation parameters of standard (reference) concrete and concrete in which fine aggregate is replaced by metallurgical sludge waste. The benefit of such concrete may be the consumption of waste material and, therefore, the creation of a more sustainable concrete mixture. Hence, it is desirable to improve, or at least maintain to some extent, similar properties as standard concrete. Some of the studied concrete mixes were enriched by air entrainment admixture to analyze its effect on the results and a superplasticizer was added to both MSW concretes. The following conclusions can be drawn from the measured data:

1. All concrete mixes show an improvement of parameters over time and to a very similar extent. The MSW does not have a negative effect on the progress of the degradation or strength parameters.
2. The compressive strength of the reference concrete with AEA is the lowest, by about 30%. The strength of MSW and MSW + AEA concrete is lower by about 8% and 15%, respectively, compared to the reference concrete. Thus, AEA in combination with MSW has a lower negative impact than the addition of AEA to the concrete alone.
3. The resistance of the MSW concrete against the aggressive substances after 91 days is about 16% lower and after 224 days is about 10% lower than the resistance of reference concrete. AEA has a slight but positive effect on the diffusion coefficient of the reference concrete. On the other hand, AEA has a negative effect on MSW concrete in terms of the diffusion coefficient, which decreases over time.

In general, it can be concluded that the combination of 30% fine aggregate replacement for MSW together with AEA shows sufficient properties for further use in construction. In the long-term research, the parameters related to the health of humans while using this material in construction as well as other degradation properties (frost resistance, etc.), need to be evaluated. Studies of the effects of metallurgical sludge are at an early stage, and the issues of potential interaction of MSW with admixtures have not yet been studied in depth. The objectives of the research presented in this article were not aimed at identifying the physicochemical interactions of MS with admixtures. Concretes with MSW can make a significant contribution to sustainability by showing the possibilities of applying waste material. Future research needs to focus on the evaluation of other parameters, the financial viability of MSW in concrete, and, last but not least, health safety.

## Figures and Tables

**Table 1:** Chemical composition of metallurgical sludge waste (%) [12].

**Fe_2_O_3_**	**SiO_2_**	**CaO**	**MgO**	**Al_2_O_3_**	**Mn**	**P_2_O_5_**	**Fe(II)**
21.85	6.65	13.56	1.34	1.65	0.60	0.19	20.91
**Na_2_O**	**K_2_O**	**Zn**	**S**	**C**	**Pb**	**Cl^−^**	**Ign. Loss.**
0.17	0.28	2.00	0.33	10.10	0.23	0.06	18.59

**Table 2:** Composition of concrete mixtures.

Label	Cement CEM I 42.5 R (kg/m^3^)	Water (kg/m^3^)	Natural Fine Aggregate (kg/m^3^)	Natural Coarse Aggregate (kg/m^3^)	Metallurgical Sludge Waste (kg/m^3^)	Superplasticizer (%/cem)	Air-Entraining Admixture (%/cem)
Reference	380	200	448	1342	0	0	0
MSW	380	200	314	1342	134	0.92	0
Ref + AEA	380	200	448	1342	0	0	0.30
MSW + AEA	380	200	314	1342	134	0.92	0.48

**Table 3:** Laboratory test results at 28 days.

Time Point	Material Characteristic			Reference	MSW	Ref + AEA	MSW + AEA
**28 days**	Density (kg/m^3^)			2345	2298	2285	2319
	Compressive strength (MPa)			55.63	51.75	42.7	48.12
	Electrical resistivity (kΩcm)	Bulk	Cylinder	5.1	4.5	5.1	4.2

**Table 4:** Laboratory test results at 56 days.

Time Point	Material Characteristic			Reference	MSW	Ref + AEA	MSW + AEA
**56 days**	Compressive strength (MPa)			60.45	56.46	45.36	51.27
	Electrical resistivity (kΩcm)	Surface	Cylinder	11.8	12.3	12.8	11.1
			Prismatic	11.6	11.3	12.8	10.7
		Bulk	Cylinder	5.6	4.9	5.6	4.6
			Prismatic	5.5	4.8	5.5	4.3
	RCPT (Coulomb)			2746	2636.5	3698	2182.5

**Table 5:** Laboratory test results at 91 days.

Time Point	Mixture Label			Reference	MSW	Ref + AEA	MSW + AEA
**91 days**	Compressive strength (MPa)			55.63	51.75	42.7	48.12
	Electrical resistivity (kΩcm)	Bulk	Cylinder	5.1	4.5	5.1	4.2

## Data Availability

Not applicable.
